# UK Medical Cannabis Registry: A Clinical Outcomes Analysis for Epilepsy

**DOI:** 10.1002/brb3.70490

**Published:** 2025-04-18

**Authors:** Isaac Cowley, Simon Erridge, Arushika Aggarwal, Lilia Evans, Madhur Varadpande, Evonne Clarke, Katy McLachlan, Ross Coomber, Augustin Iqbal, James J Rucker, Mark W Weatherall, Mikael H Sodergren

**Affiliations:** ^1^ Imperial College Medical Cannabis Research Group, Department of Surgery and Cancer Imperial College London London UK; ^2^ Curaleaf Clinic London UK; ^3^ St. George's Hospital NHS Trust London UK; ^4^ Department of Psychological Medicine Kings College London London UK; ^5^ South London and Maudsley NHS Foundation Trust London UK; ^6^ Buckinghamshire Healthcare NHS Trust Amersham UK

**Keywords:** Cannabidiol, Cannabis, Δ9‐tetrahydrocannabinol, Epilepsy

## Abstract

**Background:**

A third of epilepsy patients fail to enter seizure remission despite optimal therapeutic management. Cannabis‐based medicinal products (CBMPs) have shown promise as a potential therapy. However, a paucity of high‐quality literature regarding CBMPs’ efficacy and safety profile means further investigation is needed. The study aimed to examine changes in epilepsy‐specific and general health‐related quality of life (HRQoL) patient‐reported outcome measures (PROMs) in individuals with treatment‐resistant epilepsy.

**Methods:**

A case series of patients with epilepsy from the UK Medical Cannabis Registry analyzed changes in Quality of Life in Epilpesy‐31 (QOILE‐31), Single‐Item Sleep Quality Score (SQS), EQ‐5D‐5L, Generalized Anxiety Disorder‐7 (GAD‐7) and Patient Global Impression of Change (PGIC) between baseline, one, three, and six months. Adverse events (AEs) were collected and classified by severity. *p* < 0.050 was considered statistically significant.

**Results:**

There were 134 patients included. Improvements were recorded from baseline to one, three, and six months in QOILE‐31 and all HRQoL PROMs (*p* < 0.050). Forty patients (29.85%) reported a minimal clinically important difference in Quality of Life in Epilepsy‐31 (QOLIE‐31) at six months. There were 18 (13.43%) AEs reported by 5 (3.73%) patients, mainly mild and moderate.

**Discussion:**

The proportion of patients achieving a clinically significant change is similar to existing CBMPs in epilepsy literature. AE incidence was lower than similar studies although this may be due to the large proportion (67.16%) of individuals who were not cannabis naïve.

**Conclusion:**

Initiation of CBMPs was associated with an improvement across all PROMs. CBMPs were well tolerated across the cohort. However, randomized controlled trials are needed to help determine causality.

**Clinical Trial Registration:**

## Graphical abstract

This study assesses CBMPs) for treatment‐resistant epilepsy using UK medical cannabis registry data. Significant improvements in quality of life, anxiety, and sleep were observed, with few AE. Findings suggest CBMPs are well tolerated, but randomised trials are needed to confirm efficacy.

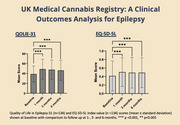

## 1 Background

Epilepsy is a chronic disease in which patients have either had at least two unprovoked seizures greather than 24 hours apart or a high risk of recurrence after one unprovoked seizure (Fisher et al. 2014). There are approximately 51.7 million people living with epilepsy, which is associated with a 2–3 times increased risk of death (Vaughan et al. 2018, Nevalainen et al. 2012, Thurman et al. 2017, Watila et al. 2018). Psychosocial factors associated with epilepsy, such as anxiety, depression, and stigma, can also result in reduced HRQoL for affected individuals (Babu et al. 2009, Tombini et al. 2021). Treatment‐resistant epilepsy (TRE) occurs in approximately one‐third of patients who fail to achieve seizure remission, despite optimal anti‐seizure medication (Yao et al. 2019, Nair 2016). As higher seizure frequency is associated with an increased risk of mortality (Nevalainen et al. 2012, Lhatoo and Sander 2005), it is of great importance to address this therapeutic gap.

The endocannabinoid system modulates the release of synaptic transmitters (Opitz et al. 2007). Endocannabinoids, such as anandamide (AEA) and 2‐arachidonoylglycerol (2‐AG), bind to presynaptic type 1 cannabinoid receptors (CB1), through retrograde signaling (Opitz et al. 2007, Cristino et al. 2020). This reduces calcium influx and the associated release of neurotransmitters, such as glutamate and γ‐aminobutyric acid (GABA) (Romigi et al. 2010, Erridge et al. 2023, Sharma et al. 2021). Several studies support the anti‐seizure effects of AEA in animal models (Al‐Hayani 2005, Khaksar et al. 2022). Furthermore, patients with temporal lobe epilepsy have reduced levels of AEA (Romigi et al. 2010). In rodent models, 2‐AG is associated with reduced seizure severity and duration (Sugaya et al. 2016). Endocannabinoids also produce anticonvulsant effects through CB1‐independent mechanisms (Sugaya et al. 2016). Conversely, these two endocannabinoids have also been shown to have proconvulsant effects, mediated via transient receptor potential vanilloid type 1 (TRPV1) channels (De Petrocellis and Di Marzo 2009).

CBMPs have emerged as a therapeutic category for epilepsy treatment. Cannabidiol (CBD) is derived from *Cannabis sativa* L. and has been shown to have anti‐seizure properties (Lattanzi et al. 2018). The complete mechanism is unclear; however, CBD is an endocannabinoid modulator (Gaston and Szaflarski 2018). CBD inhibits AEA reuptake and hydrolysis, producing anticonvulsant effects (Gaston and Szaflarski 2018). CBD further affects epileptogenesis through blockade of human T‐type calcium channels and sodium channels (Gaston and Szaflarski 2018). CBD also has a complex relationship with TRPV1, whereby it can lead to concentration‐dependent activation or inhibition of the channel, leading to pro‐ or anticonvulsant effects, respectively (Iannotti et al. 2014, De Petrocellis et al. 2011).

Epidyolex, a CBD oil isolate, is licensed as an adjunctive medication for TRE secondary to dravet syndrome, lennox‐gastaut syndrome, and tuberous sclerosis complex (NICE 2023, NICE 2021). A meta‐analysis of four large randomized controlled trials demonstrated a significant decrease in seizure frequency compared to placebo in patients treated with CBD (Devinsky et al. 2020). A recent meta‐analysis also found CBD increased the number of individuals reporting a ≥ 50% reduction in seizure frequency by 20% (Silvinato et al. 2022).

Many people, however, continue to experience seizures despite optimization of CBD dose (Devinsky et al. 2020, Silvinato et al. 2022). Therefore, there is interest in exploring broad‐spectrum CBMPs, which also include other phytocannabinoids, such as Δ^9^‐tetrahydrocannabinol (Δ^9^‐THC) (Nair 2016) (McPartland et al. 2015). There is conflicting evidence in animal models regarding whether Δ^9^‐THC has pro‐ or anticonvulsant effects (Appendino et al. 2019, 2017, Li et al. 2023). Interactions between CBD and Δ^9^‐THC, when administered together in broad‐spectrum CBMPs, have been shown to be complex. For example, CBD has been shown to affect the metabolism of Δ^9^‐THC, increasing plasma Δ^9^‐THC levels (Bornheim et al. 1993). Conversely, CBD has been identified as a noncompetitive negative allosteric modulator of CB1 (Beers et al. 2023). This reduces the efficacy of agonists, like Δ^9^‐THC, which bind to CB1 (Chung et al. 2019). There is limited evidence on Δ^9^‐THC in isolation in epilepsy, with most literature assessing its effects within the context of broad spectrum CBMPs. An Australian study found that in people with epilepsy who used cannabis, 89.5% reported it to be beneficial in helping their epilepsy (Suraev et al. 2017). An American study of 225 patients, meanwhile, reported that 75% achieved a reduction in seizures with CBMP oils (Sulak et al. 2017). One in ten of these patients achieved complete seizure remission (Sulak et al. 2017). Furthermore, a study in Israel using broad‐spectrum CBMPs in children with TRE showed that 89% of patients achieved a decrease in seizure frequency (Tzadok et al. 2016). A previous UK Medical Cannabis Registry (UKMCR) study in children with TRE, found 94.1% of participants prescribed broad‐spectrum CBMPs reported a > 50% reduction in monthly seizures, with no change in AEs when compared to the CBD isolate arm of the study (Erridge et al. 2023). Currently, there are unknown risks associated with long‐term CBMP administration considering the paucity of high‐quality randomized controlled trials in the treatment of epilepsy. The primary aim of this study was to therefore assess the change in PROMs of patients enrolled in the UKMCR who are treated with CBMPs for epilepsy. The secondary aim was to assess the prevalence of AEs within the cohort of patients prescribed CBMPs for epilepsy.

## 2 Methods

### 2.1 Study Design

This case series analyzed patients with epilepsy enrolled in the UKMCR for a minimum of six months prior to December 13 2023. All patients were provided with information about the UKMCR and asked to provide informed consent prior to their initial appointment. Patients were therefore, enrolled consecutively. For those not prescribed CBMPs after their initial consultation, their data were censored and removed from the UKMCR. The UKMCR has approval from the Central Bristol Research Ethics Committee (ref. 22/SW/0145). This study was reported following the STrengthening the Reporting of OBservational studies in Epidemiology (STROBE) guidelines.

### 2.2 Setting and Participants

The UKMCR, which is owned by Curaleaf Clinic, was created in 2019 and is responsible for collecting, recording and pseudonymizing data from patients treated with CBMPs in the UK (Olsson et al. 2023). Following UK guidelines, all patients prescribed CBMPs had to have failed to receive sufficient benefit from licensed anti‐seizure medications prior to initiating CBMPs (Case 2020). Therefore, by definition, all patients enrolled had TRE. CBMP options included medium‐chain triglyceride oils (or reformulations of the oil), dried flower, or both. All CBMPs supplied to patients were grown in conditions that met good agricultural and collection practices and manufactured into the end product in accordance with good manufacturing practice (MHRA 2020). All unlicensed CBMPs must conform to standards outlined by the European Pharmacopoeia, and the assurances that CBMPs meet these standards must be confirmed by the Medicines and Healthcare products Regulatory Agency (MHRA 2020).

The inclusion criteria were for patients who were enrolled in the UKMCR with a primary indication for treatment of epilepsy. Patients with secondary and tertiary indications for treatment with CBMPs were still included in the study if their primary indication was epilepsy. The exclusion criteria included patients who had not filled out any baseline PROMs.

### 2.3 Data Collection

Upon enrollment, patients provide demographic information, including age, height, weight, gender, occupation, and medication. The Charlson Comorbidity Index, a validated way of giving insight into a patient's long‐term risk of mortality due to comorbidities (Charlson et al. 2022), was calculated by clinicians in initial consultations. Smoking status, pack years, and alcohol consumption were assessed. Lifetime cannabis use was assessed using the measure of “gram years,” calculated by multiplying grams used per day by the number of years of consumption.

CBMP data were collected from prescription data, including route of administration and dosage of CBD and Δ^9^‐THC. CBMP dosages were calculated by taking the concentration (mg/g) of each respectively and multiplying by the prescribed daily dose of oil/flower.

### 2.4 Outcome Measures

Primary outcome measures were changes in PROMs from baseline to one, three, and six months. Secondary outcome measure included the incidence of AEs and their severity.

PROMs collected for patients in this study were Quality of Life in Epilepsy‐31 (QOLIE‐31), EuroQoL‐5 Dimensions‐5 Levels (EQ‐5D‐5L), Single‐Item Sleep Quality Scale (SQS), Generalized Anxiety Disorder‐7 (GAD‐7), and Patient Global Impression of Change (PGIC). All PROMs, other than PGIC, had baselines collected upon registration and were completed remotely (Tait et al. 2023).

The QOLIE‐31 questionnaire has seven domains, including seizure worry, overall quality of life, emotional well‐being, energy/fatigue, medication effects, social function, and cognitive function. Each domain is ranked on a scale of 0–100 and combined to give an overall score of 0–100 (Barbara 2024). The greater the overall score, the greater the overall HRQoL. Cognitive function is given the greatest weighting in the final score, with medication effects the lowest. The mean score for QOLIE‐31, among a sample representative of the general epilepsy population, is 63 (Barbara 2024). The minimal clinically important difference (MCID) change for QOLIE‐31 is five (Borghs et al. 2012). QOLIE‐31 has been shown to be reliable and valid in providing information on HRQoL in epilepsy (Cramer et al. 1998). The questionnaire takes approximately 20–25 minutes to complete (Kumar et al. 2023).

The ED‐5D‐5L is a standardized tool for assessing HRQoL and is recommended by the National Institute for Health and Care Excellence (NICE) (2009). Ranked on a scale of “one” (no problem) to “five” (extreme problem), there are five domains: mobility, self‐care, pain/discomfort, usual activity, and anxiety/depression. An overall index score is produced, with scores of 1 implying perfect HRQoL and scores less than zero implying a HRQoL worse than death (van Hout et al. 2012).

The SQS is a single question that measures sleep quality over the last week on a scale of 0 (terrible) to 10 (excellent). The SQS has been shown to be reliable and valid when compared to longer forms of sleep assessment, such as the Pittsburgh Sleep Quality Index (Dereli and Kahraman 2021).

The GAD‐7 assesses symptoms of generalized anxiety, with seven questions covering the core characteristics of GAD and their impact from “zero” (not at all) to “three” (nearly every day). Overall scores, out of 21, give a classification from mild (≥5), moderate (≥10) and severe (≥15) anxiety (Terlizzi and Villarroel 2019). GAD‐7 has a strong internal consistency and is a reliable measure for GAD when compared to validated measuring tools (Johnson et al. 2019, Guzick et al. 2024).

The PGIC assesses the patient's perceived change since starting treatment, on a scale from “one” (no change or worsening condition) to “seven” (a great deal better).

Patients could report AEs contemporaneously during the event utilizing a bespoke online platform. Participants were also prompted to report any AEs that had not been reported prior to completion of PROMs, Finally, clinicians could also input AEs directly into the UKMCR from the patient's electronic healthcare record. AE severity was recorded in line with the common terminology criteria for AEs version 4.0 (National Institutes of Health 2009).

### 2.5 Statistical Analysis

Baseline demographics and current CBMPs were analyzed using descriptive analysis. They are displayed as the mean (± standard deviation), median (interquartile range), and frequency (%) as appropriate. AEs are listed and presented as proportions of participants.

To assess the primary outcome, a repeated‐measures analysis of variance (ANOVA) was performed. Values of statistical significance were analyzed with a post‐hoc pairwise comparison with Bonferroni correction, to mitigate against a type I error. With a sufficient study size, PROM data were considered parametric, in line with the central limit theorem. In the case of missing follow‐up PROMs, baseline observations carried forward (BOCF) were applied.

Univariate and multivariate logistic regressions were performed to assess variables for association with an MCID in the mean QOILE‐31 scores at six months. The outcomes of these were presented as odds ratios (ORs) with 95% confidence intervals (95% CIs).

Statistical analysis was performed using Statistical Package for the Social Sciences (SPSS) [IBM Statistics version 29, SPSS (New York, Illinois), USA]. GraphPad Prism (v. 9.4.1(350)) was used to produce graphs. Statistical significance was determined as *p* < 0.050.

## 3 Results

At the time of data extraction (December 13 2023) from the UKMCR, there were 19,763 patients enrolled, of which 134 (0.68%) were included in this study. Reasons for exclusion include lack of completion of baseline PROMs (*n* = 1105; 5.59%), enrolled in the UKMCR less than 6 months prior to data extraction (*n* = 5,910; 29.90%), and epilepsy not being the primary indication for therapy (*n* = 12,614; 63.83%).

### 3.1 Patient Demographics

The mean age of patients was 36.87 (± 12.00) years (**Table** **1**). The mean body mass index of the cohort was 25.81 (±6.89) kg/m^2^. Most patients were male (n = 91; 67.91%). The median Charlson Comorbidity Index score was 0.00 (0.00‐0.25).

**TABLE 1:** Patient baseline demographic data and tobacco, alcohol, and cannabis status at enrolment. This includes sex, age, body mass index (BMI), occupation by category, charlson comorbidity index, tobacco status, pack years, alcohol intake, cannabis status, daily cannabis use, gram years and use of cannabis frequency. Abbreviations: *n*: Number of participants; %: Percentage; *SD*: Standard deviation; IQR: Interquartile range. (*n* = 134).

Demographics		*n* (%) / mean ± *SD*. / median [IQR]
Gender	Female	43 (32.09%)
	Male	91 (67.91%)
Age (years)		36.87 ± 11.99
BMI (kg/m^2^)		25.81 ± 6.89
Occupation	Armed forces occupations	1 (0.75%)
	Clerical support workers	1 (0.75%)
	Craft and related trades workers	3 (2.24%)
	Elementary occupations	5 (3.73%)
	Managers	2 (1.49%)
	Other occupations	23 (17.16%)
	Plant and machine operators, and assembles	1 (0.75%)
	Professional	8 (5.97%)
	Service and sales workers	6 (4.48%)
	Skilled agricultural, forestry, and fishery workers	1 (0.75%)
	Technicians and associate professionals	4 (2.99%)
	Unemployed	72 (53.73%)
	Unknown	7 (5.22%)
Charlson comorbidity index		0.00 [0.00‐0.25]
Tobacco status	Current Smoker	39 (29.10%)
	Ex‐smoker	44 (32.84%)
	Never smoked	51 (38.06%)
Pack years		8.00 [2.00‐20.00]
Weekly alcohol consumption (units)		0.00 [0.00‐0.00]
Cannabis status	Current user	90 (67.16%)
	Ex‐user	14 (10.45%)
	Never used	30 (22.39%)
Daily cannabis usage (grams)		1.50 [1.00‐2.00]
Lifetime quantity of cannabis consumed (gram years)		5.50 [2.00‐20.00]
Cannabis use frequency	Every day	77 (85.56%)
	Every other day	7 (7.78%)
	1‐2 times a week	5 (5.56%)
	Greater than 1 time a month	1 (1.11%)

### 3.2 Tobacco, Alcohol and Cannabis Status

Patient data regarding tobacco, alcohol, and cannabis status is displayed in **Table** **1**. Eighty‐three (61.94%) participants were current or ex‐smokers, with a median pack year history of 8.00 [2.00‐20.00]. Thirty (22.39%) participants were cannabis naïve. Median cannabis gram years for current and ex‐users (*n* = 104, 77.61%) were 5.50 [Δ^9^‐THC 2.00 to 20.00] grams.

### 3.3 Prescription Cannabis‐Based Medicinal Products

CBMP prescription data was available for all patients (n = 134, **Table** **2**). At baseline, median total dose of CBD was 17.50 (1.00 to 30.00) mg/day, and Δ^9^‐THC was 6.50 (1.00 to 19.50) mg/day. At six months, the median total dosage of CBD was 50.00 (10.00 to 90.00) mg/day and Δ9‐THC was 93.90 (5.88 to 135.16) mg/day. At baseline, 61 (45.52%) patients used oil sublingually, 41 (30.60%) used dry flower for vaporization, and 32 (23.88%) both. At six months, there was a shift to patients using both forms of CBMP with 51 (38.06%) patients using oil, 40 (29.85%) using dry flower and 43 (32.09%) both. Adven EMC1 50/< 4 mg/ml CBD/Δ9‐THC (Curaleaf International, United Kingdom). and Adven EMT 20 mg/ml Δ9‐THC (curaleaf international, United Kingdom) were the most frequently prescribed CBD‐ dominant oils and Δ9‐THC‐dominant oils. The most commonly prescribed flos was Adven EMT2 16% < 1% Δ9‐THC/CBD (curaleaf international, United Kingdom).

**TABLE 2:** Data on prescribed CBMPs (*n* = 134). This includes prescription information represented as median [interquartile range (IQR)], and administration routes, represented as counts (%). *Two participants removed from CBMP analysis at one and three months to prevent inadvertent re‐identification (*n* = 132). CBD—Cannabidiol; THC—Δ^9^‐tetrahydrocannabinol.

n (%)/ median [IQR]				
	Baseline	Follow up at one month	Follow up at three months	Follow up at six months
Administration*				
Oils	61 (45.52%)	58 (43.28%)	55 (41.04%)	51 (38.06%)
Dry flower	41 (30.60%)	38 (28.36%)	38 (28.36%)	40 (29.85%)
Oils and dry flower	32 (23.88%)	37 (27.61%)	39 (29.10%)	43 (32.09%)
Dosage (milligrams/day)				
CBD	17.50 [1.00 ‐ 30.00]	47.50 [5.00 ‐ 80.00]	36.25 [10.00–81.25]	50.00 [10.00 ‐ 90.00]
Δ9‐THC	6.50 [1.00–19.50]	48.55 [4.88 ‐100.00]	81.08 [5.00–125.00]	93.90 [5.88–135.16]

### 3.4 Patient Reported Outcome Measures

PROM scores throughout the study period are displayed in **Table** **3**. Pairwise comparisons between statistically significant PROMs on repeated‐measures ANOVA are displayed in **Table** **4**.

**TABLE 3:** Epilepsy‐specific and general health‐related quality of life (HRQoL) patient reported outcome measures (PROMs) at baseline, one, three, and six months. Changes assessed by repeated measures analysis of variance (ANOVA). Quality of Life in Epilepsy‐31 (QOLIE‐31) (*n* = 123), EuroQoL‐5 Dimensions‐5 Levels (EQ‐5D‐5L) (*n* = 133), Generalized Anxiety Disorder‐7 (GAD‐7) (*n* = 134), Single‐Item Sleep Quality Scale (SQS) (*n* = 132) and Patient Global Impression of Change (PGIC) (*n* = 101). *p*‐values shown. Data displayed as mean ± standard deviation.

PROMs	Baseline	1 month	3 months	6 months	*p*‐value
QOLIE‐31	38.99 ± 16.30	48.93 ± 20.24	47.75 ± 19.27	46.37 ± 20.13	< 0.001
Overall quality of life score	44.07 ± 18.80	51.71 ± 20.01	51.01 ± 20.18	48.93 ± 19.86	< 0.001
Seizure worry scale	25.28 ± 25.39	35.38 ± 29.52	35.72 ± 28.94	34.58 ± 31.48	< 0.001
Social functioning scale	33.44 ± 24.40	45.14 ± 29.22	44.63 ± 27.76	43.69 ± 27.02	< 0.001
Cognitive functioning scale	42.13 ± 24.11	53.83 ± 26.13	51.36 ± 25.41	49.91 ± 27.50	< 0.001
Medication effects scale	27.26 ± 29.93	35.46 ± 33.59	36.09 ± 31.67	34.92 ± 32.10	< 0.001
Emotional wellbeing scale	47.81 ± 21.70	56.74 ± 22.81	54.19 ± 22.81	53.42 ± 24.58	< 0.001
Energy fatigue scale	36.77 ± 18.47	43.95 ± 21.37	44.15 ± 20.85	42.02 ± 21.24	< 0.001
EQ‐5D‐5L Index Value	0.41 ± 0.35	0.50 ± 0.34	0.49 ± 0.36	0.49 ± 0.35	< 0.001
GAD‐7	10.43 ± 6.87	8.04 ± 6.61	8.28 ± 6.64	8.54 ± 6.72	< 0.001
SQS	4.11 ± 2.57	5.31 ± 2.54	5.08 ± 2.72	4.80 ± 2.81	< 0.001
PGIC		4.94 ± 1.65	5.35 ± 1.44	5.36 ± 1.40	< 0.001

**TABLE 4:** Pairwise comparison of epilepsy‐specific and general health‐related quality of life (HRQoL) patient reported outcome measures (PROMs). p‐values shown. Pairwise comparison including baseline, one month, three months and six months for all sets of data other then Patient Global Impression of Change (PGIC) which has no baseline. Quality of Life in Epilepsy‐31 (QOLIE‐31) (*n* = 123), EuroQoL‐5 Dimensions‐5 Levels (EQ‐5D‐5L) (*n* = 133), Generalized Anxiety Disorder‐7 (GAD‐7) (*n* = 134), Single‐Item Sleep Quality Scale (SQS) (*n* = 132) and PGIC (*n* = 101). Data displayed as mean difference (MD) ± standard deviation (SD).

	Baseline		1 month		3 months	
QOLIE‐31 Total	MD ± SD	*p*‐value	MD ± SD	*p*‐value	MD ± SD	*p*‐value
One month	−9.94 ± 1.41	< 0.001				
Three months	−8.76 ± 1.24	< 0.001	1.19 ± 0.96	1.00		
Six months	−7.38 ± 1.32	< 0.001	2.57 ± 1.10	0.126	1.38 ± 0.93	0.850
EQ‐5D‐5L Index	MD ± SD	*p*‐value	MD ± SD	*p*‐value	MD ± SD	*p*‐value
One month	−0.90 ± 0.21	< 0.001				
Three months	−0.75 ± 0.17	< 0.001	0.02 ± 0.02	1.00		
Six months	−0.76 ± 0.17	< 0.001	0.02 ± 0.02	1.00	0.00 ± 0.02	1.00
GAD‐7	MD ± SD	*p*‐value	MD ± SD	*p*‐value	MD ± SD	*p*‐value
One month	2.39 ± 0.47	< 0.001				
Three months	2.14 ± 0.40	< 0.001	−0.25 ± 0.33	1.00		
Six months	1.89 ± 0.40	< 0.001	−0.50 ± 0.40	1.00	−0.25 ± 0.29	1.00
SQS	MD ± SD	*p*‐value	MD ± SD	*p*‐value	MD ± SD	*p*‐value
One month	−1.21 ± 0.22	< 0.001				
Three months	−0.98 ± 0.19	< 0.001	0.23 ± 0.19	1.00		
Six months	−0.69 ± 0.19	0.003	0.52 ± 0.20	0.068	0.29 ± 0.16	0.443
PGIC	MD ± SD	*p*‐value	MD ± SD	*p*‐value	MD ± SD	*p*‐value
One month						
Three months			−0.41 ± 0.12	0.004		
Six months			−0.42 ± 0.12	0.001	−0.01 ± 0.09	1.00

There was an improvement in QOLIE‐31 total mean score (*n* = 123) from baseline to one, three, and six months (*p* < 0.001) and this improvement was further seen in all seven domains of the QOILE‐31. Whilst a decrease in mean score can be seen following month one, this change was not significant (*p* > 0.050).

An improvement from baseline in overall HRQoL was noted following one, three, and six months (all *p* < 0.001) as shown by the change in EQ‐5D‐5L index (*n* = 133). No change was noted within three of the five domains from baseline to month one (mobility (*p* = 1.00), self‐care (*p* = 0.549) and usual activities (*p* = 0.145)). Symptoms of generalized anxiety improved across one, three, and six months from baseline (*p* < 0.001), as measured by GAD‐7 (*n* = 134). SQS (*n* = 132) showed an increase in sleep quality at one month (*p* < 0.001), three months (*p* < 0.001), and six months (*p* = 0.003) from baseline.

PGIC scores at month one, three, andsix, were 4.94 (±1.65), 5.35 (±1.43), and 5.36 (±1.40) respectively. There was an increase from month one to month three (*p* = 0.004) and month six (*p* = 0.001).

### 3.5 Adverse Events

Eighteen AEs (13.43%) were reported by five patients (3.73%). The most frequent AEs were delirium, fatigue, and headache, each of which had an incidence of two (1.49%) (**Table** **5**). There were no life‐threatening AEs. One patient withdrew and ceased taking CBMPs due to their the adverse events they experienced. Current users, ex‐users, and cannabis naïve individuals all reported AEs.

**TABLE 5:** Prevalence and severity of adverse events (AEs) reported during the study. AEs (*n* = 18) are categorized by severity; mild, moderate, or severe as reported by patients (*n* = 5).

Adverse events	Mild	Moderate	Severe	Total (%)
Cognitive disturbance	1	0	0	1 (0.75%)
Concentration impairment	1	0	0	1 (0.75%)
Confusion	0	1	0	1 (0.75%)
Delirium	0	2	0	2 (1.49%)
Dizziness	1	1	0	2 (1.49%)
Dry mouth	1	0	0	1 (0.75%)
Fatigue	0	2	0	2 (1.49%)
Headache	0	2	0	2 (1.49%)
Insomnia	1	0	0	1 (0.75%)
Lethargy	1	0	0	1 (0.75%)
Mood swings	1	0	0	1 (0.75%)
Nausea	1	0	0	1 (0.75%)
Seizure	0	1	0	1 (0.75%)
Somnolence	0	0	1	1 (0.75%)
Total	8 (5.97%)	9 (6.72%)	1 (0.75%)	18 (13.43%)

### 3.6 Univariate and Multivariate Analysis

Univariate analysis results can be seen in **Table** **6**. Univariate analysis analyzed variables for their relationship with the MCID in QOLIE‐31 scores. Forty patients (29.85%) achieved the MCID for QOLIE‐31 at 6 months. Univariate analysis showed that those who were treated with dried flower only increased odds for a meaningful positive change (OR = 2.71, 95% CI = 1.05‐6.96; *p* = 0.039). Below median Δ^9^‐THC dose (< 93.90 mg/day) was associated with reducing the chance of a positive change (OR = 0.44, 95% CI = 0.20‐0.95; *p* = 0.037) in univariate analysis.

**TABLE 6:** Univariate logistic regression of odds ratios for age, gender, body mass index (BMI), route of cannabis‐based medicinal product (CBMP) administration, cannabis status at baseline, cannabidiol (CBD) dose, and Δ^9^‐tetrahydrocannabinol (Δ^9^‐THC) dose. Results displayed as odds ratio (OR) (95% confidence interval (CI)).

Variable		*n*	OR (95% CI)	*p*‐value
Age (years)	18‐24	17	1.00	
	25‐34	40	2.77 (0.69‐11.12)	0.152
	35‐44	32	1.83 (0.42‐7.91)	0.421
	45‐54	22	2.18 (0.47‐10.12)	0.321
	55+	10	7.00 (1.19‐41.36)	0.32
Gender	Female	40	1.00	
	Gender	84	0.88 (0.40‐1.95)	0.752
BMI (kg/m^2^)	18.50‐24.99	56	1.00	
	0.00‐18.49	10	0.71 (0.17‐3.07)	0.651
	25.00‐29.99	33	0.83 (0.34‐2.06)	0.693
	30.00‐34.99	9	1.33 (0.32‐5.53)	0.692
	35+	11	0.37 (0.07‐1.88)	0.231
Route of CBMPs administration	Oil	45	1.00	
	Dried flower	39	2.71 (1.05‐6.96)	0.039
	Both	40	1.89 (0.72‐4.91)	0.194
Cannabis Status	Never used	27	1.00	
	Current user	83	1.62 (0.61‐4.27)	0.331
	Ex‐user	14	1.14 (0.27‐4.84)	0.856
CBD dose	Below median	63	1.00	
	Above median	61	1.59 (0.75‐3.39)	0.228
Δ^9^‐THC dose	Below median	59	1.00	
	Above median	65	0.44 (0.20‐0.95)	0.037

Multivariate analysis results can be seen in **Table** **7**. Multivariate analysis showed that those aged 55+ years old had increased odds for a meaningful positive change in QOLIE‐31 score (OR = 8.59, 95% CI = 1.17‐62.87; *p* = 0.034). No other factors of BMI, gender, route of CBMP administration, cannabis status on enrolment, CBD and Δ^9^‐THC dose were associated with a change in odds.

**TABLE 7:** Multivariate logistic regression of odds ratios for age, gender, body mass index (BMI), route of CBMP administration, cannabis status at baseline, (CBD dose and (Δ^9^‐THC dose. Results displayed as odds ratio (OR) (95% confidence interval (CI)).

Variable		*n*	OR (95% CI)	*p*‐value
Age (years)	18‐24	17	1.00	
	25‐34	40	3.13 (0.69‐14.21)	0.140
	35‐44	31	2.17 (0.46‐10.35)	0.330
	45‐54	21	2.56 (0.48‐13.79)	0.274
	55+	10	8.59 (1.17‐62.87)	0.034
Gender	Female	40	1.00	
	Gender	79	0.86 (0.3‐2.28)	0.763
BMI (kg/m^2^)	18.50‐24.99	56	1.00	
	0.00‐18.49	10	1.70 (0.31‐9.33)	0.542
	25.00‐29.99	33	1.10 (0.41‐2.93)	0.856
	30.00‐34.99	9	1.48 (0.29‐7.717)	0.639
	35+	11	0.38 (0.07‐2.08)	0.266
Route of CBMPs administration	Oil	44	1.00	
	Dried flower	39	1.26 (0.27‐5.99)	0.769
	Both	36	1.02 (0.20‐5.21)	0.986
Cannabis Status	Never used	26	1.00	
	Current user	79	0.87 (0.23‐3.35)	0.837
	Ex‐user	14	0.58 (0.11‐3.11)	0.520
CBD dose	Below median	62	1.00	
	Above median	57	1.02 (0.41‐2.56)	0.963
Δ^9^‐THC dose	Below median	58	1.00	
	Above median	61	2.55 (0.62‐10.44)	0.194

## 4 Discussion

This observational case series assessed the change in PROMs and AEs in individuals treated with CBMPs for TRE. The results suggest an association between initiating CBMP treatment and an improvement in patient‐reported epilepsy‐specific outcomes, alongside improvements in anxiety, sleep quality, and general HRQoL. With 96.27% of patients not reporting any AEs, this suggests that CBMPs are generally well‐tolerated.

There were increases in mean QOLIE‐31 scores at all points following baseline. This increase in mean score was one of clinical significance, with 29.85% of patients attaining the MCID at six months. This is similar to the 33% of patients who achieved > 50% reduction in seizure frequency following treatment with oral cannabis extracts (Press et al. 2015). A > 50% reduction is frequently used in anti‐seizure medication trials as an endpoint and deemed to be of clinical significance (Birbeck et al. 2002). Whilst this present study did not collect seizure number, the overall score for QOLIE‐31 has been shown to be indirectly correlated to seizure number (Cramer et al. 1998). Given this and the fact that overall seizure worry decreased (Baseline; 25.28 ± 25.39 to six months: 34.58 ± 31.48) it can be estimated that seizure frequency also improved in this study, but not proven. A retrospective study in the United States reporting on non‐prescription cannabis found a greater than 50% reduction in 55% of their cohort, higher than the rate in the present study but still comparable (Tzadok et al. 2016). The multivariate analysis showed that above the median Δ^9^‐THC daily doses were associated with a reduced OR for achieving a MCID in QOLIE‐31. This is supported with data from Erridge et al., where the greatest seizure reductions seen were in the treatment arm receiving Δ^9^‐THC alongside CBD (Erridge et al. 2023). However, the Δ^9^‐THC doses in this study were lower than those reported in the present study. As previously outlined, Δ^9^‐THC has demonstrated both anti‐ convulsant and pro‐convulsant effects in laboratory studies (Li et al. 2023, Bornheim et al. 1993). Therefore, further assessment of the optimal dose of Δ^9^‐THC compared to CBD is required. This is particularly important considering recent observational data on isolated CBD showing over one‐third of individuals with TRE prescribed CBD isolate experience a > 50% reduction in seizure frequency (Kühne et al. 2023 Jun). As this analysis showed the reduction was independent of epilepsy subtype it will be important to understand in which patients adjunctive therapy with full‐spectrum CBMPs, including Δ9‐THC, is warranted.

There was an improvement in self‐reported sleep quality from baseline to 1, 3, and 6, months. Sleep quality is not only an important component of HRQoL, but moreover poor sleep quality is a risk factor for increased seizure frequency, seizure burden and disease progression (Bonilla‐Jaime et al. 2021, Roitman et al. 2014). The effects of CBMP on sleep are not fully understood, with some studies stating that Δ^9^‐THC at doses as low as 10 mg/day may increase sleep quality, whilst others reported increased cannabis use decreased overall sleep quality (Roitman et al. 2014, Maple et al. 2016, Lavender et al. 2022, Wahby et al. 2019). Considering this and that the mechanism of action of CBMPs is incompletely understood it is important to consider whether changes observed are secondary to off‐target effects, rather than a direct anticonvulsant effects.

The decrease in GAD‐7 score is indicative of a change of the mean population value from “mild” to “subclinical” generalized anxiety. This disagrees with the findings of Whaby et al., who found that cannabis use was associated with depression and reduced HRQoL (Wahby et al. 2019). The source of the cannabis in this study, however, was not standardized, with some participants obtaining it illegally, which is a confounding factor. In the present study, CBMPs had to meet regulatory requirements of consistency and safety, and care was overseen by a clinical team (MHRA 2020). In contrast, illicit cannabis may be contaminated with other substances which may be anxiogenic (Seely et al. 2012). In addition, there is less guidance over the optimum dose strategy. At sufficiently high doses Δ^9^‐THC can be anxiogenic (Lichenstein 2022). Finally, engaging in illicit activity may also increase individual anxiety (Couch 2022). In the present study, 67.16% were consuming illicit cannabis at baseline and transferred to CBMPs. This may have also contributed to reductions in reported anxiety.

Improvements were also observed in the EQ‐5D‐5L index, yet this was not seen in all five domains. Only pain/discomfort and anxiety/depression demonstrated significant change from baseline to one month. Patients from Canada demonstrated similar increases in HRQoL following cannabis treatment (Cahill et al. 2021). Similar to the present study, most patients were male and greater than 50% of patients had previous cannabis use (Cahill et al. 2021). Patients did not experience a significant improvement in their mobility or self‐care, which may be due to epilepsy not impacting these domains sufficiently to be detected using this measure.

Five patients reported AEs, which had an overall prevalence of 18 (13.43%). This is similar to a meta‐analysis looking at CBD oil with dosage ranging from 5–50 mg/kg/day for 3–16 weeks which reported 9.7% for the CBD group and 4.0% for control (Fazlollahi et al. 2023). However, another similar study using CBD and Δ9^9^‐THC reported a 34.5% for their rate of AEs (Devinsky et al. 2022). It should be considered that this trial had participants on a maximum dose of 300 mg/day CBD and 6 mg/day Δ^9^‐THC, which is different from the final dose at six months of 50.00 (IQR: 10.00‐90.00) mg/day CBD and 93.90 (5.88‐135.16) mg/day Δ^9^‐THC. The present study's AEs prevalence may be lower than expected given the large predominance of current users of cannabis at baseline. These individuals may have developed tolerance to the effects of cannabis or different interpretations of the psychoactive effects of CBMPs, which leads them to not report these as AEs (Gorelick et al. 2013). Finally, there was no assessment by clinicians to determine whether all AEs were treatment‐related. Therefore, the AEs reported in this study may not necessarily be secondary to CBMPs, but also symptoms of underlying disease or concomitantly prescribed medications. Furthermore, with a median baseline CBD and Δ^9^‐THC dose of 17.50 (1.00‐30.00) mg/day and 6.50 (1.00‐19.50) mg/day respectively to CBD and Δ^9^‐THC of 50.00 (10.00‐90.00) mg/day and 93.90 (5.88‐135.16) mg/day, it is clear that the medications have been titrated over time. Through adopting a titration regimen, this may help limit the incidence of AEs.

### 4.1 Limitations

Given the observational design of this study, it is impossible to demonstrate causality between CBMPs and improved epilepsy‐specific and HRQoL PROMs. The lack of blinding may lead to over reporting changes in PROMs. Moreover, CBMPs are associated with an increased placebo effect and expectancy bias compared to other medications (Gedin et al. 2022).

This may be further exacerbated by selection bias. Due to the nature of a private clinic having self‐selecting patients, patients with prior improvement following use of cannabis may have been more likely to register. Given the context that most patients (77.61%) were current or ex‐users, which is larger than the hypothesized lifetime prevalence in people with epilepsy of between 10–40%, this may have contributed to the positive results observed (Beers et al. 2023). Conversely, these individuals may have already developed pharmacological tolerance to cannabis, limiting the effect CBMPs may have (Uliel‐Sibony et al. 2021). Furthermore, there is a greater prevalence of men (67.9%) than women within the study, despite women being more likely to develop TRE (Cepeda et al. 2022). This could be because men are more likely than women to use illicit cannabis (Hamerle et al. 2014, Lekoubou et al. 2020). Future assessment comparing cannabis naïve individuals against current users at baseline would help elucidate further the effect of prior cannabis use on reported outcomes. Finally, with the study concluding at six months, this limits the ability to assess long term CBMP use regarding its AEs profile, tolerance, and long‐term efficacy. PROMs are subjective and may be influenced by recall bias.

### 4.2 Conclusion

Treatment with CBMPs was associated with an improvement in both epilepsy‐specific and general HRQoL outcomes at one, three, and six months. This study shows the promising potential of CBMPs as an adjunctive treatment option in the management of TRE. However, this study has its own limitations and as a result it is hard to deduce causality and as such future randomized control trials are needed to fully evaluate the viability of broad‐spectrum CBMPs in TRE.

## Author Contributions

**Isaac Cowley**: Writing–original draft, Writing–review and editing, Formal analysis, Methodology, Data curation, Project administration. **Simon Erridge**: Conceptualization, Methodology, Data curation, Investigation, Formal analysis, Supervision, Writing–original draft, Writing–review and editing, Project administration. **Arushika Aggarwal**: Data curation, Methodology, Writing–original draft, Writing–review and editing. **Lilia Evans**: Methodology, Data curation, Writing–review and editing, Writing–original draft. **Madhur Varadpande**: Methodology, Data curation, Writing–original draft, Writing–review and editing. **Evonne Clarke**: Methodology, Data curation, Writing–original draft, Writing–review and editing. **Katy McLachlan**: Methodology, Data curation, Writing–original draft, Writing–review and editing. **Ross Coomber**: Conceptualization, Methodology, Investigation, Data curation, Writing–original draft, Writing–review and editing. **Augustin Iqbal**: Writing–original draft, Writing–review and editing, Data curation, Investigation. **James J Rucker**: Data curation, Investigation, Conceptualization, Methodology, Writing–original draft, Writing–review and editing. **Mark W Weatherall**: Conceptualization, Methodology, Data curation, Investigation, Writing–original draft, Writing–review and editing. **Mikael H Sodergren**: Conceptualization, Methodology, Data curation, Investigation, Formal analysis, Supervision, Project administration, Resources, Writing–original draft, Writing–review and editing.

## Ethical approval

The UK Medical Cannabis Registry and derivative research studies have been afforded a favourable ethical opinion by the Health Research Authority (South West—Central Bristol Research Ethics Committee reference 22/SW/0145).

## Consent

All participants completed written, informed consent prior to enrolment in the registry. Permission to reproduce material from other sources: N/A

## Funding

There was no external or commercial funding associated with this paper.

## Conflicts of Interest

SE, EC, KC, RC, AI, JJR, MWW and MHS are employed by or work on a consultancy basis for Curaleaf Clinic.

### Peer Review

The peer review history for this article is available at https://publons.com/publon/10.1002/brb3.70490

## Data Availability

The data that support the findings of this study are available from UK Medical Cannabis Registry. Restrictions apply to the availability of these data, which were used under license for this study. Data are available from the author(s) with the permission of UK Medical Cannabis Registry.
